# Macrophage-fibroblast signaling networks identified by single-cell RNA sequencing in juvenile systemic sclerosis

**DOI:** 10.1172/jci.insight.202801

**Published:** 2026-06-23

**Authors:** Aneri Shethji, Theresa Hutchins, Anwesha Sanyal, Tianhao Liu, Wei Chen, Kathryn S. Torok

**Affiliations:** Department of Pediatrics, University of Pittsburgh, Pittsburgh, Pennsylvania, USA.

**Keywords:** Dermatology, Immunology, Autoimmune diseases, Fibrosis, Rheumatology

## Abstract

<style type=”text/css”>a { text-decoration: none; color: #464feb; } tr th, tr td { border: 1px solid #e6e6e6; } tr th { background-color: #f5f5f5; } </style> Single‑cell analysis of juvenile systemic sclerosis skin reveals altered fibroblast–macrophage interactions that may drive fibrosis and identify pathways relevant to pediatric disease.

**To the Editor:** Juvenile-onset systemic sclerosis (jSSc) is a rare and severe fibrosing autoimmune disease with high morbidity, yet the cellular mechanisms driving tissue fibrosis in children remain undefined. To address this gap, we performed single-cell RNA sequencing (scRNA-seq) on jSSc skin to identify disease-specific stromal and immune subclusters and define multicellular signaling networks underpinning pediatric fibrosis. We focus on fibroblast-macrophage interactions, consistent with a macrophage predominance within collagen-dense dermal lesions in juvenile (1) and adult scleroderma (2).

Single-cell profiling of jSSc skin (*n* = 9) compared with healthy pediatric skin (*n* = 9) revealed distinct fibroblast and macrophage subsets (Figure 1, A and B, Supplemental Figure 1A and Supplemental Table 1; supplemental material available online with this article; https://doi.org/10.1172/jci.insight.202801DS1). Among 11 fibroblast subclusters, several populations were enriched in jSSc (Figure 1B). *COL11A1^+^POSTN^+^* fibroblasts expressing *COL11A1*, *COL1A2*, *COL5A1*, and *POSTN* (Supplemental Table 2) mirrored a subcluster identified in adult SSc (3) and juvenile localized scleroderma (4), with strong *TGFB*, *WNT*, and *FGF* activation by IPA (Supplemental Figure 1C), consistent with a stiffness-responsive, matrix-producing myofibroblast phenotype. This *COL11A1* population was prominent in an adult SSc scRNA-seq meta-analysis (*n* = 182), correlating with histopathologic fibrosis and myofibroblast markers (*ACTA2*, *CTGF*, and *TAGLN*) (5).

We additionally identified a previously unrecognized *STC1^+^FZD9^+^* fibroblast population absent from adult SSc scRNA-seq datasets and significantly increased in jSSc, suggesting a pediatric-specific fibrotic endotype (Supplemental Figure 1B and Supplemental Table 2). IPA demonstrated expression of pathogenic fibroblast programs seen in pulmonary and hepatic fibrosis, *STC1*, *FZD9*, *SFRP2*, *MYH9*, and oxidative stress genes (Supplemental Figure 1D). Enrichment of *TGFB*, *WNT*, *IGF*, metabolic, and mitochondrial stress pathways suggested a phenotype adapted for sustained matrix turnover, with *FZD9* implicating non-canonical *WNT* signaling and *STC1* suggesting altered oxygen sensing.

Macrophage clustering identified 10 transcriptionally distinct subsets, several enriched in jSSc skin (Figure 1, C and D). *MMP19^+^CTSL^+^* macrophages (Supplemental Figure 1B and Supplemental Table 3) expressed *MMP19*, *CTSL*, and *CTSB*, consistent with matrix-remodeling activity and release of matrix-bound growth factors that facilitate fibroblast infiltration. While the *MMP19^+^CTSL^+^* macrophage cluster is not upregulated in the disease state, its disease relevance is reflected by amplified ligand-target activity in Nichenet analyses. *NOTCH*-polarized macrophages expressing *NOTCH2NLB* and *NOTCH2NLC* further supported broad polarization toward fibrotic activation (Supplemental Figure 1E), with similar heightened *NOTCH* signatures in jSSc fibroblasts (Supplemental Figure 1F) (6).

CellChat analysis demonstrated that macrophage-fibroblast interactions constituted the core signaling axis in jSSc. Diseased skin exhibited divergent interaction strength and pathway diversity relative to healthy controls, particularly involving *TGFB* pathways, with communication between *MMP19^+^CTSL^+^* macrophages and *STC1^+^FZD9^+^* fibroblasts observed only in jSSc (Figure 1E). This dense, bidirectional communication network suggested a self-reinforcing macrophage-fibroblast circuit (Supplemental Figure 1G).

NicheNet ligand-target analysis identified macrophages as the dominant upstream sender cells to fibroblasts within the jSSc signaling ecosystem (Figure 1F). Macrophage-derived ligands, including *TGFB1*, *AREG*, *IL1B*, *COL6A2*, *SPP1*, and *JAG1*, were strong predicted drivers of fibroblast gene programs associated with both the *COL11A1^+^POSTN^+^* and *STC1^+^FZD9^+^* clusters. Corresponding fibroblast receptors (*TGFBR1*, *TGFBR2*, *FGFR1*, *FGFR2*, *IL1R1*, *NOTCH2*, *FZD9*, and *ITGAV*) matched upregulated receptor expression in the scRNA-seq data, supporting the activation of core profibrotic signaling pathways. *WNT* pathway genes reinforced the association between *FZD* receptors and non-canonical *WNT* signaling. Modeling also suggested a bidirectional circuit in which macrophages initiated strong *TGFB* and *TNF* inputs into fibroblasts, while fibroblasts amplified *WNT*, *IGF*, and *NOTCH* signals that fed back into macrophages and neighboring fibroblasts.

Spatial transcriptomic profiling (10X Visium HD) provided in situ validation of these predicted interactions (Figure 1G). Across 3 pilot jSSc biopsies, we observed discrete lesional niches where fibroblast subsets, *STC1^+^FZD9^+^*, *SFRP2^+^SFRP4^+^PCOLCE2^+^*, and *ANGPTL7^+^*, colocalized with macrophages (Figure 1, G and H). In these niches, fibroblasts upregulated genes associated with an inflammatory microenvironment, such as *CXCL12*, *C3*, and *CD74* (Supplemental Figure 1, H–J). These observations demonstrate that macrophage-fibroblast signaling hubs are spatially organized within jSSc lesions and likely serve as focal engines of fibrosis.

In summary, these findings reveal a pediatric-specific multicellular fibrotic circuit (Figure 1I) defined by pathogenic macrophage and fibroblast subsets, including a previously unrecognized *STC1^+^FZD9^+^* fibroblast population and spatially organized macrophage-fibroblast niches. Persistent *TGFB* activation emerged as a unifying feature across both cell types. Upregulation of *TGFBR1*, *TGFBR2*, *ACVR1B*, and *ENG* (Figure 1F) supported canonical *SMAD2*/3 signaling, consistent with the *COL11A1^+^* fibroblast population in adult SSc where heightened *SMAD3* regulon activity correlates with histologic and clinical skin fibrotic burden (3). The convergence of macrophage-derived ligands (*TGFB1*, *AREG*, *COL6A2*, *JAG1*) on fibroblast receptors, together with persistent integrin-mediated *TGFB* activation, suggests a self-reinforcing signaling loop. These interconnected pathways highlight potential therapeutic nodes for disrupting macrophage-fibroblast circuits in jSSc.

Detailed methods, including statistical analyses, study approval, data availability, and acknowledgments, are included in the Supplemental Methods.

## Conflict of interest

The authors have declared that no conflict of interest exists.

## Funding support

This work is the result of NIH funding, in whole or in part, and is subject to the NIH Public Access Policy. Through acceptance of this federal funding, the NIH has been given a right to make the work publicly available in PubMed Central.

- National Institute of Arthritis and Musculoskeletal and Skin Diseases (NIAMS) grants R01 AR086434 (to KT and WC) and P50 AR060780 (to KT).
- Department of Defense USAMRDC grant W81XWH-21-1-0782 (to KT).
- Scleroderma Research Foundation awards (to KT and WC).

## Supplementary Material

Supplemental data

Supplemental tables 1-3

Supporting data values

## Figures and Tables

**Figure 1:**
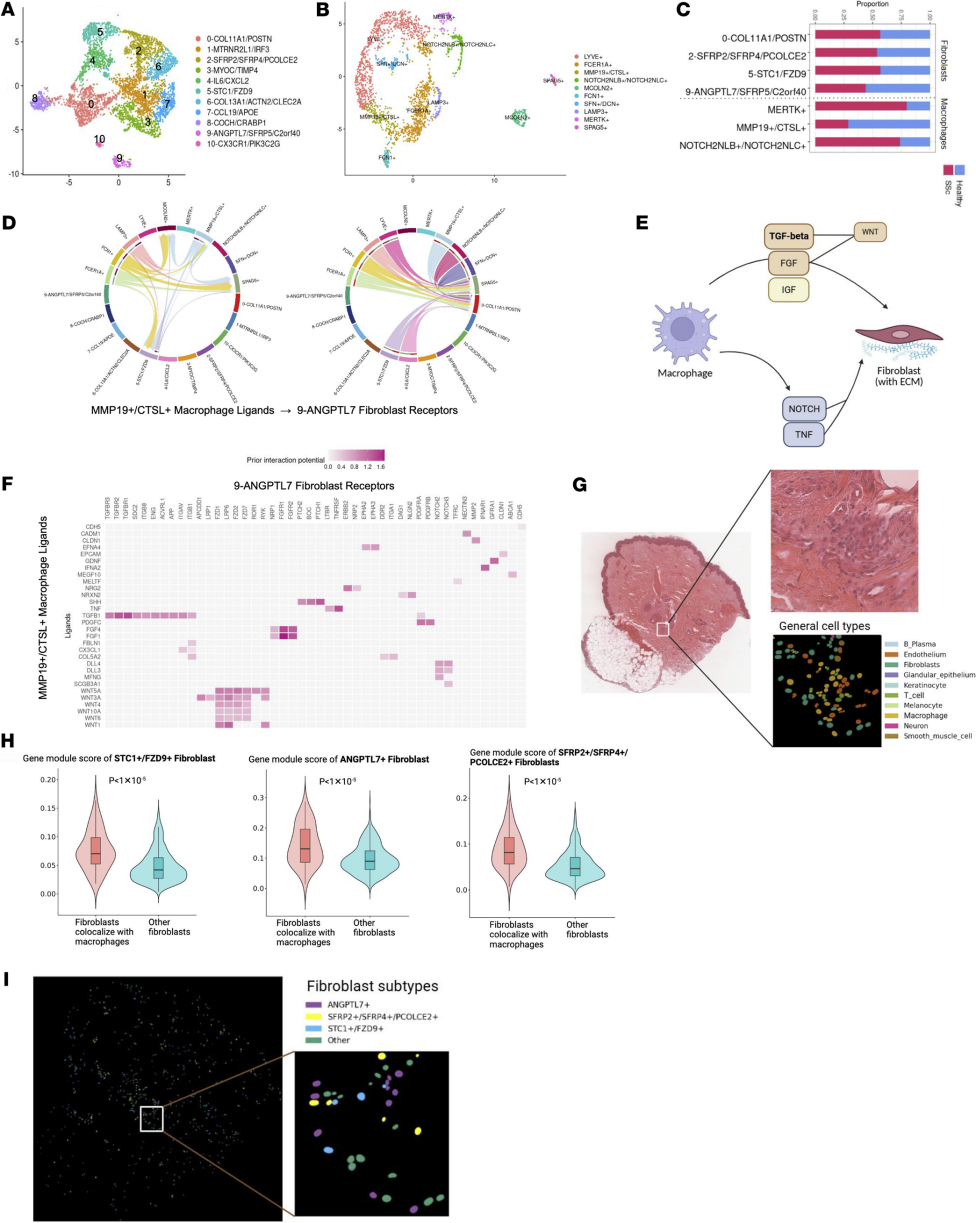
Skin single-cell analysis identifies pathogenic macrophage-fibroblast circuits in juvenile-onset systemic sclerosis (jSSc). (**A**) UMAP of 8,860 skin fibroblasts (4,893 jSSc and 4,893 healthy) identifying 11 transcriptionally distinct subclusters by marker gene expression. (**B**) UMAP of 2,719 macrophages (852 jSSc and 1,867 healthy) identifying 10 annotated subclusters. (**C**) Bar plot of fibroblast and macrophage subcluster proportions in jSSc versus healthy samples. (**D**) CellChat analysis of *TGFB* ligand-receptor interactions between fibroblast and macrophage subtypes in jSSc (left) and healthy (right) skin, showing expanded intercluster connectivity in jSSc; numbered clusters denote fibroblasts, unnumbered denote macrophages. (**E**) Schematic of a macrophage-driven profibrotic circuit: macrophage-derived *TGFB*, *FGF*, and *IGF* activate fibroblast ECM programs via *WNT* signaling, while *NOTCH* and *TNF* reinforce chronic fibroblast activation. (**F**) NicheNet interaction heatmap highlighting signaling from *MMP19*^+^*CTSL*^+^ macrophages to *ANGPTL7*^+^ fibroblasts via ligands (*TGFB1*, *AREG*, and *COL6A2*) and receptors (*TGFBR1*, *FGFR1*, and *FZD9I*). (**G**) Spatial transcriptomics showing macrophage-fibroblast colocalization (<25 μm) in jSSc skin; insets highlight high-resolution regions in the reticular dermis, perivascular infiltrate region. (**H**) Marker genes of *STC1*^+^*FZD9*^+^, *ANGPTL7*^+^, and *SFRP2*^+^*SFRP4*^+^ were significantly upregulated in the fibroblasts that colocalized with macrophages compared with non-colocalized fibroblasts (*P* < 1 × 10^–5^ using the Wilcoxon rank-sum test; Bonferroni-adjusted *P* < 0.05). (**I**) Spatial localization of *STC1*^+^*FZD9*^+^, *ANGPTL7*^+^, and *SFRP2*^+^*SFRP4*^+^ fibroblasts in the same biopsy region as shown in **G**.

## Version 1 06/23/2026

In-Press Preview

## Version 2 08/10/2026

Electronic publication
