# Supplementary material for: Macrophage-fibroblast signaling networks identified by single-cell RNA sequencing in juvenile systemic sclerosis
Source: JCI Insight. 2026 Jun 23;11(15):e202801. doi: 10.1172/jci.insight.202801 (PMC13463615; doi:10.1172/jci.insight.202801)
Supplement: Supplementary file 1 [file jciinsight-11-202801-s151.pdf]

Supplemental Material

Macrophage–Fibroblast Signaling Network Identified by Single-Cell RNA Sequencing in Juvenile Systemic Scleroderma

Methods

Sex as a Biological Variable

Samples for the single-cell dataset were obtained from both male (n = 5) and female (n = 13) participants. Samples for the spatial transcriptomic dataset were also obtained from both male (n = 1) and female (n = 2) participants. Sex was recorded at collection but not analyzed separately, as sex-based differences were not the focus of this study and the number too small.

Patient and Skin biopsy Details

This dataset is composed of 4-mm area punch biopsy samples of the affected area from SSc patients or forearm and chest from healthy controls who consented to participate in either of the following research study protocols- National Registry of Childhood Onset Scleroderma (NRCOS) (the University of Pittsburgh #STUDY19080297), Connective Tissue Disease (CTD) Registry (University of Pittsburgh, #STUDY19090054), and Morphea in Adults and Children (MAC) Registry (University of Texas Southwestern, #STU112010-028) or Skin Bx for Research (University of Pittsburgh - STUDY19090212). All 9 jSSc participants enrolled in this study are

pediatric onset patients (onset of <18 years of age). Healthy controls were age and sex-matched to the 9 SSc patients whenever possible.

Single-Cell RNA Sequencing

Fresh or CryoStor® CS10 (BioLife Solutions®, Bothell, WA, USA) preserved samples were processed, as described in our earlier publication (1). Biopsy samples were enzymatically digested (Miltenyi Biotec Whole Skin Dissociation Kit, human Cat# 130-101-540 Miltenyi Biotec®, Gaithersburg, MD, USA); Miltenyi gentleMACS Octo Dissociator (Cat# 130-096-427, Miltenyi Biotec®, Gaithersburg, MD, USA). The suspended cells were filtered using 70-µm cell strainers and re-suspended in 0.04% BSA+ PBS. Post filtration, the cells were mixed with reverse transcription reagents and loaded into the Chromium instrument (10x Genomics®, Pleasanton, CA, USA), a commercial application of Drop-Seq (2). The Chromium instrument then formed Gel bead emulsions - GEMs - oligonucleotide that is composed of an oligo-dT section for priming reverse transcription, and barcodes for each cell (10x Genomics®) and each transcript (unique molecular identifier, UMI) (3). Approximately 4000 cells were loaded per sample. V1, V2, and V3 single-cell chemistries were used in accordance with the manufacturer's protocol (10x Genomics). Post reverse transcription, the emulsion was broken using a recovery agent, and following Dynabead and SPRI clean up, cDNAs were amplified by 11–12 cycles of PCR (C1000, Bio-Rad, Hercules, CA, USA). cDNAs were sheared (Covaris, Woburn, MA, USA) into ~200 bp length. The library was quantified using a KAPA Universal Library Quantification Kit KK4824 (Roche Diagnostics Corporation, Indianapolis, IN, USA) and further characterized for cDNA length on a bioanalyzer using a high-sensitivity DNA kit(Illumina, San Diego, CA, USA). Libraries

were sequenced (~200 million reads/sample), using the Illumina NextSeq-500 platform (Illumina, San Diego, CA, USA).

Spatial Transcriptomics

Visium Spatial Gene Expression (10x Genomics, Pleasanton, CA, USA) was run on formalin-fixed paraffin-embedded (FFPE) skin biopsy from three jSSc patients. The tissue sections were taken from FFPE tissue block and analyzed on Visium HD (2μm spots) platform from 10x Genomics. The slide cassette of Visium HD captured 6.5 mm × 6.5 mm capture area. The raw sequencing data and images were processed with 10x Genomics Space Ranger 3.1.2. The transcriptomics data and the Hematoxylin and Eosin (H&E) stained image were merged and aligned by the spatial coordinate of the slide. The cells were segmented with StarDist (4) and gene expression of 2μm spots were aggregated into single cells. To annotate the cell types in spatial transcriptomics data, we used the paired gene expression in single-cell RNA sequencing data (scRNA-seq) data from the same patients (adjacent biopsy) as reference. The cell types manually labeled in scRNA data were transferred to cells in spatial transcriptomics data with RCTD (5). The RCTD annotated cells in spatial transcriptomics data with the gene expression of in both scRNA data and spatial transcriptomics data and information of spatial locations (6). The colocalization between fibroblasts and macrophages (distance < 25μm) were identified in some niches. The expression of gene modules for different subtypes of fibroblasts or macrophages were compared between the cells in the colocalized niches and other fibroblasts.

Data Preprocessing and Bioinformatics Analyses

All FASTQ files obtained post sequences underwent CellRanger processing according to the proper protocol based on the sequencing type. Sequencing reads were examined by quality metrics, and transcripts were mapped to reference human genome (GRCh38). Cell Ranger (10x Genomics, Pleasanton, CA, USA) was used to assign reads to particular cells according to their barcode. Sequencing data was pulled together and aggregated in a single object, and layers were merged via Seurat for further analysis. The data from this study is deposited on NCBI Gene Expression Omnibus (GSE320020; prior healthy control data: GSE264508, GSE138669, GSE288490).

Sample analyses were performed using R (version 4.5.0) packages. Seurat 5.0 (7) package was specifically used for normalization and filtering, gene expression analysis, cell type identification and to utilize other supporting packages including NicheNet, ggpubr and CellChat. The data was subsetted by the parameters of nFeature_RNA < 4000, percent.mt < 50, percent. ribo < 50 and nCount_RNA < 20000. Genes were filtered according to the Filter Genes function, with a minimum value of 0.5 and minimum cells of 100. Principal component analysis (PCA) was performed on the highly variable genes and Harmony was used to integrate the dataset based on the library_id (sample ID) (8). This data was further clustered and visualized by UMAP with dims set to 1:20. Clustering was performed on the data using the "FindNeighbors" function with dimensions set to 1:20 and using the "harmony" reduction previously ran. Clusters were identified using the FindClusters function with a resolution set to 0.6 using codes adapted from the Saltija lab (9). Cell types were identified based on these clusters, and were identified using a combination of known marker genes (10, 11) used in our previous publications as well as top differentially expressed genes for each cluster. Although the total number of fibroblasts included for analysis was equivalent between jSSc and control samples, this reflects analytical filtering and

normalization steps rather than comparable fibroblast abundance. Fibroblasts and macrophages were further investigated by sub-setting and re- clustering them out from the original Seurat object.

Cell-Communication Analysis via NicheNet and CellChat

Seurat wrapper packages were used for further analysis of cell communication pathways and gene interactions. Following cell subclustering, fibroblast and macrophage populations were subset from the processed scRNA-seq dataset for communication analysis. The disease state was used as a parameter for all further analysis. CellChat (12) was used to predict the most upregulated interactions amongst fibroblast and macrophage subtypes when comparing the disease state to the control state. Interactions enriched in the jSSc disease state relative to healthy controls were prioritized to identify signaling pathways with increased predicted activity. CellChat analyses were performed using the curated ligand–receptor interaction database provided by the package. NicheNet (13) was used to further investigate these interactions based on the CellChat results, as NicheNet provides more nuanced information target genes and ligand interaction. Both packages utilize literature supported databases consisting of ligand-target interaction pathways, signal transduction, and gene regulatory interactions.

Statistical Analysis

Differential gene expression (DGE) between jSSc and healthy samples within each cell cluster and subcluster was performed using the Libra (14) package's edgeR-based pseudobulk methodology. Bonferroni correction was applied to adjust p-values (significance threshold < 0.05), and differential expression was reported as log2 fold change (log2FC), with an absolute

$log2FC \geq 1$ considered significant. Differentially expressed genes were visualized using the Enhanced Volcano package.

Ingenuity pathways analysis (IPA) (15), was applied to DEG lists to identify enriched upstream regulators and canonical pathways in jSSc fibroblasts relative to healthy controls. For comparisons of fibroblast and macrophage subcluster RNA counts, statistical significance was assessed using two-tailed unpaired t tests ($p < 0.05$).

For spatial transcriptomics, gene expression in fibroblasts located within macrophage-enriched regions was compared with fibroblasts outside these regions using Wilcoxon rank sum test with Bonferroni-adjusted $p < 0.05$.

All statistical analyses were performed using R and Seurat (v4.4.0), and differences were considered statistically significant at $p < 0.05$.

Study Approval

All tissues were collected and analyzed under Institutional Review Board approval using the following research study protocols with appropriate consent per study: the National Registry of Childhood Onset Scleroderma (NRCOS) (the University of Pittsburgh #STUDY19080297), Connective Tissue Disease (CTD) Registry (University of Pittsburgh, #STUDY19090054), and Morphea in Adults and Children (MAC) Registry (University of Texas Southwestern, #STU112010-028) or Skin Bx for Research (University of Pittsburgh - STUDY19090212).

Data Availability Statement

All data relevant to the study is readily available in supplementary documents, including "Supportive data values" excel for data points shown in graphs and means of Figure 1H and Supplementary Figures 1B and 1H and "Cell counts and proportions by subject" excel. Single-cell RNA-seq datasets have been deposited on NCBI Gene Expression Omnibus (GSE number GSE320020, GSE264508, GSE138669, GSE288490). Additional data is available upon request (Kathryn Torok; kathryn.torok@chp.edu).

Acknowledgments

The authors thank Megan Natali, Dr. Michael Bykowski, and the Pediatric Plastic Surgery Division at UPMC Children's Hospital of Pittsburgh for their assistance with tissue acquisition and skin biopsy procedures for both jSSc and age-matched control participants. We also acknowledge Dr. Heidi Jacobe (University of Texas Southwestern) for providing a pediatric healthy control specimen and Dr. Robert Lafyatis (University of Pittsburgh) for contributing two young adult healthy control skin samples and as director of the University of Pittsburgh Single Cell Core, which was utilized for this study. We are deeply grateful to the patients with juvenile systemic sclerosis and their families at the University of Pittsburgh Medical Center whose participation made these single-cell and spatial transcriptomic analyses possible.

Author Contributions Statement

Conceptualization: KT, AS[2]; Formal Analysis: AS[1], TH, TL; Funding Acquisition: KT, WC; Investigation: KT, AS[1], AS[2], TH, TL, WC; Methodology: KT, AS[2], TH, WC; Project Administration: KT; Supervision: KT, WC; Visualization: AS[1], TH, TL; Writing – Original Draft Preparation: AS[1], TH, KT; Writing – Review and Editing: AS[2], KT, TH, WC

AS[1] – Aneri Shethji; AS[2] – Anwesha Sanyal

Supplemental References:

1. *Mirizio, E., et al., Single-cell transcriptome conservation in a comparative analysis of fresh and cryopreserved human skin tissue: pilot in localized scleroderma. Arthritis Res Ther, 2020. 22(1): p. 263.*
2. *Macosko, E.Z., et al., Highly Parallel Genome-wide Expression Profiling of Individual Cells Using Nanoliter Droplets. Cell, 2015. 161(5): p. 1202-1214.*
3. *Zheng, G.X., et al., Massively parallel digital transcriptional profiling of single cells. Nat Commun, 2017. 8: p. 14049.*
4. *Schmidt, U., et al. Cell detection with star-convex polygons. in International conference on medical image computing and computer-assisted intervention. 2018. Springer.*
5. *Cable, D.M., et al., Robust decomposition of cell type mixtures in spatial transcriptomics. Nat Biotechnol, 2022. 40(4): p. 517-526.*
6. *Liu, T., et al., Spatial Transcriptomics Identifies Cellular and Molecular Characteristics of Scleroderma Skin Lesions: Pilot Study in Juvenile Scleroderma. Int J Mol Sci, 2024. 25(17).*

7. *Hao, Y., et al., Dictionary learning for integrative, multimodal and scalable single-cell analysis. Nat Biotechnol, 2024. 42(2): p. 293-304.*

8. *Korsunsky, I., et al., Fast, sensitive and accurate integration of single-cell data with Harmony. Nat Methods, 2019. 16(12): p. 1289-1296.*

9. *Satija, R., et al., Spatial reconstruction of single-cell gene expression data. Nat Biotechnol, 2015. 33(5): p. 495-502.*

10. *Hutchins, T., et al., Characterization of Endothelial Cell Subclusters in Localized Scleroderma Skin with Single-Cell RNA Sequencing Identifies NOTCH Signaling Pathway. Int J Mol Sci, 2024. 25(19).*

11. *Werner, G., et al., Single-Cell Transcriptome Analysis Identifies Subclusters with Inflammatory Fibroblast Responses in Localized Scleroderma. Int J Mol Sci, 2023. 24(12).*

12. *Jin, S., M.V. Plikus, and Q. Nie, CellChat for systematic analysis of cell-cell communication from single-cell transcriptomics. Nat Protoc, 2025. 20(1): p. 180-219.*

13. *Browaeys, R., W. Saelens, and Y. Saeys, NicheNet: modeling intercellular communication by linking ligands to target genes. Nat Methods, 2020. 17(2): p. 159-162.*

14. *Squair, J.W., et al., Confronting false discoveries in single-cell differential expression. Nat Commun, 2021. 12(1): p. 5692.*

15. *Kramer, A., et al., Causal analysis approaches in Ingenuity Pathway Analysis. Bioinformatics, 2014. 30(4): p. 523-30.*

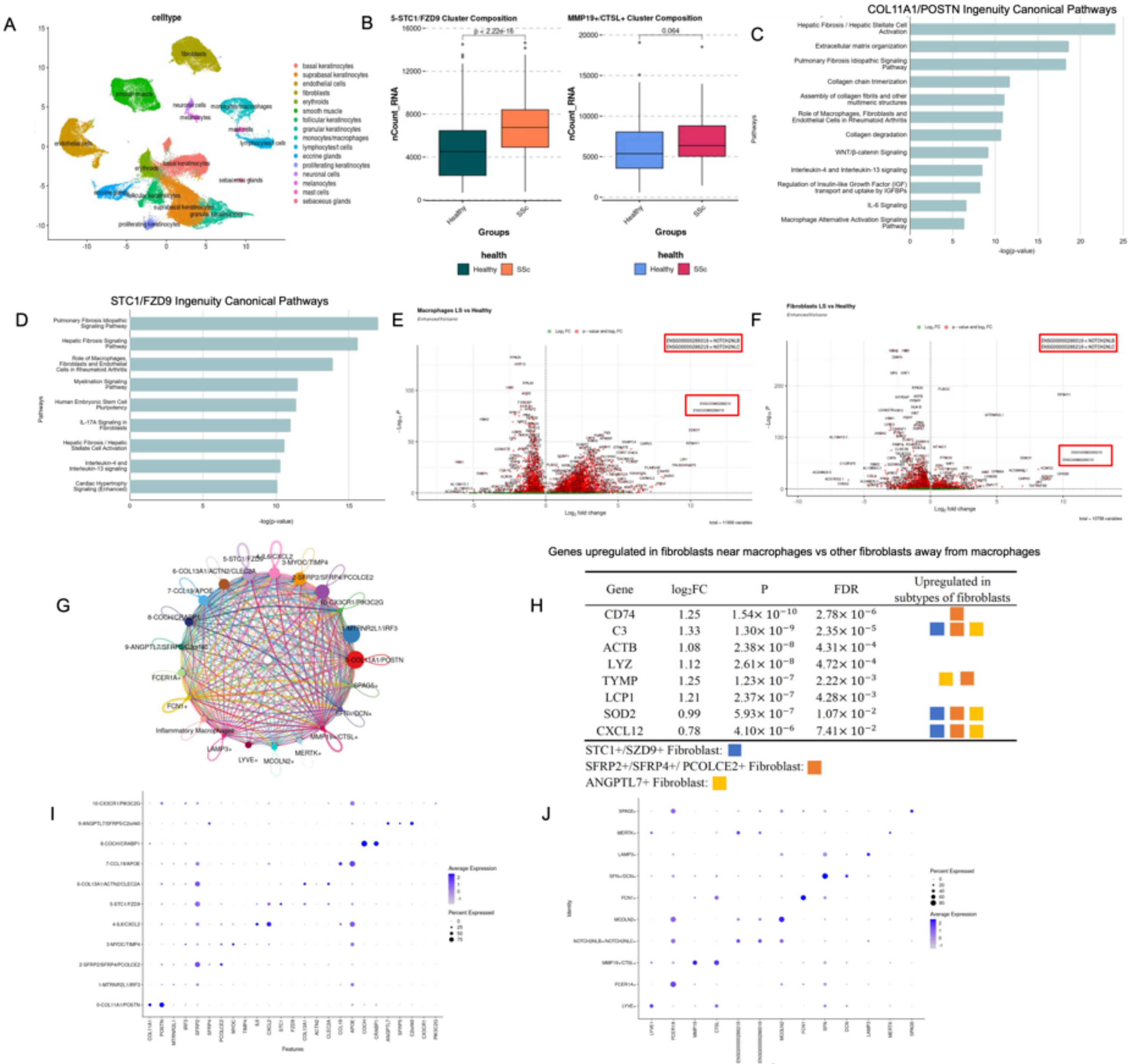

Supplementary Figure Legend

Supplemental Figure 1:

Data derived from scRNA-seq and Visium HD spatial transcriptomics of lesional jSSc skin.

(A) UMAP plot of all cells (65,645 total: 32,014 jSSc and 33,631 healthy cells) showing cell-type annotations across all jSSc and healthy skin samples, highlighting major populations including fibroblasts, macrophages, T cells, and endothelial cells. **(B)** Boxplots show total transcript counts (nCount_RNA) for the $STC1^{+}/FZD9^{+}$ fibroblast cluster (left) and the $MMP19^{+}/CTSL^{+}$ macrophage cluster (right) in healthy pediatric skin and juvenile SSc (jSSc) skin. The $STC1^{+}/FZD9^{+}$ cluster is significantly expanded in SSc ($p < 2.22 \times 10^{\square 1 \square}$), while the $MMP19^{+}/CTSL^{+}$ cluster shows a trend toward higher abundance in SSc ($p = 0.064$), using Wilcoxon rank sum test with Bonferroni-adjusted $p < 0.05$. **(C)** Ingenuity Pathway Analysis of the COL11A1+/POSTN+ fibroblast cluster revealing pathways associated with Collagen-expressing genes, FGF, WNT, and interleukin signaling. **(D)** Ingenuity Pathway Analysis of the novel STC1+/FZD9+ fibroblast cluster revealing enrichment of profibrotic signaling pathways, including pulmonary and hepatic fibrosis signaling and TGF-beta pathway activation. **(E)** Volcano plots depicting differentially expressed genes between healthy and disease-state macrophages. Each point represents one gene, plotted by its logarithmic fold change (x-axis) and negative log-base-10 of the p value (y-axis). Significantly regulated genes appear as red points. NOTCH2NLB (ENSG00000286019) and NOTCH2NLC (ENSG00000286219) are highlighted on the far right of each plot, showing strong upregulation with high statistical significance. **(F)** Volcano plots showing differentially expressed genes between healthy and disease-state fibroblasts analyzed in same manner as macrophages. NOTCH2NLB (ENSG00000286019) and NOTCH2NLC (ENSG00000286219) are highlighted on the far right of each plot, showing strong upregulation with high statistical significance. **(G)** Chord diagram with Cellchat software illustrating the number of interactions among fibroblast and macrophage cell populations. Each node represents a distinct labeled cell cluster. The thickness and color of the connecting lines convey the frequency and strength of cell–cell interactions.

Larger node size reflects a higher total number of interactions involving that cluster. **(H)** *Inflammatory genes significantly upregulated in the fibroblasts spatially colocalized with macrophages compared with non-proximal, identified using Wilcoxon rank sum test with Bonferroni-adjusted $p < 0.05$.* **(I-J)** *Dot plots showing marker gene expression across transcriptionally defined fibroblast* **(I)** *and macrophage* **(J)** *subclusters. Rows correspond to cell subclusters and columns to canonical marker genes used for annotation; dot size represents the proportion of cells expressing each gene, and color intensity indicates mean expression level.*
